# Supplementary material for: Electrocardiogram‐gated coronary CT angiography dose estimates using ImPACT
Source: J Appl Clin Med Phys. 2016 Jul 8;17(4):342–56. doi: 10.1120/jacmp.v17i4.6218 (PMC5690042; doi:10.1120/jacmp.v17i4.6218)

Electrocardiogram-gated coronary CT angiography dose estimates using ImPACT

**Abstract:** The primary study objective was to assess radiation doses using a modified form of the Imaging Performance Assessment of Computed Tomography (CT) scanner (ImPACT) patient dosimetry for cardiac applications on an Aquilion ONE ViSION Edition scanner, including the Ca score, target computed tomography angiography (CTA), prospective CTA, continuous CTA/cardiac function analysis (CFA), and CTA/CFA modulation. Accordingly, we clarified the CT dose index (CTDI) to determine the relationship between heart rate (HR) and X-ray exposure. As a secondary objective, we compared radiation doses determined using modified ImPACT, a whole-body dosimetry phantom study, and the k-factor method to verify the validity of the dose results obtained with modified ImPACT. The effective doses determined for the reference person [4.66 mSv at 60 beats per minute (bpm) and 33.43 mSv at 90 bpm] were less than approximately 10% of those determined for the phantom study (5.28 mSv and 36.68 mSv). The effective doses according to the k-factor (0.014 mSv·mGy^−1^·cm^−1^; 2.57 mSv and 17.10 mSv) were much smaller than those obtained with the other two methods. In the present study, we have shown that ImPACT, when modified for cardiac applications, can assess both absorbed and effective doses. The results of our dose comparison indicate that modified ImPACT dose assessment is a promising and practical method for evaluating coronary CTA.

Key words: computed tomography, ImPACT, effective dose, organ dose, ICRP

PACS number: 87.53.LY, 87.57.Q-, 87.57.-s

**I. Introduction**

Coronary computed tomography angiography (CCTA) is considered a reliable practical diagnostic method. An estimated 410,000 examinations are performed annually at 1535 cardiology and/or cardiovascular surgery hospitals in Japan.^(1)^ Nevertheless, CCTA has risk about the potentially high radiation dose received by patients. According to a 2008 report by the United Nations Scientific Committee on the Effects of Atomic Radiation (UNSCEAR), therefore, effective doses range of CCTA from 3 to 27.5 mSv.^(2)^ This effective dose range is wide rather than other CT examinations: head; 0.9 to 7.9 mSv, chest; 2.2 to 12.9 mSv, abdomen; 3.1 to 16.1 mSv．In addition, the effective dose of CCTA is high rather than the others, therefore, appropriate dose management is need. These effective doses were determined using a number of estimation methods, including a whole-body dosimetry phantom study with a thermoluminescent dosimeter (TLD)^(3,4)^ and a number of dosimetric application software studies such as the ImpactDose CT application (VAMP GmbH, Erlangen, Germany) with standardized male and female anthropomorphic mathematical phantoms.^(5,6)^ The phantom study represents a sophisticated clinical method. However, the absorbed and effective doses are obtained easily with the ImpactDose application, as the software can select cardiac scan protocols from a “User-specified” or “SOMATOM Definition Flash scanner” during CCTA examinations.^(7)^ Although the ImpactDose has enabled better estimations of effective doses using the ICRP publication 110 reference male and female phantoms, the relationships between heart rate (HR) and cardiac scan protocols have not yet been clarified. In contrast, the Society of Cardiovascular Computed Tomography (SCCT) has published CCTA guidelines to facilitate reliable estimations of practical effective doses.^(8-10)^ In the SCCT guidelines,^(4)^ the dose–length product (DLP), which has accordingly been defined by a number of international organizations, including the International Commission on Radiological Protection (ICRP),^(11,12)^ European Commission,^(13,14)^ and International Electrotechnical Commission.^(15-17)^, has been recommended as the most useful parameter for dose estimation. Because k-factor introduced in the 2004 European Commission guidelines as a reasonable method for estimating the effective dose is define as dividing the effective dose by the DLP of the adult chest (0.014 mSv·mGy^−1^·cm^−1^) was useful.^(13)^ However, this k-factor represents a change from the value of 0.017 mSv·mGy^−1^·cm^−1^ reported in the 2000 guidelines^(14)^ because the value of effective dose was evaluated by new tissue weighting factor. As this reason, k-factor may be revised. Moreover, a study by Andrew^(18)^ has better elucidated the cardiac k-factor (0.027–0.034 mSv·mGy^−1^·cm^−1^), which is important because the adult chest k-factor tended to underestimate the effective dose for CCTA. However, the k-factor is limited by its lack of assessment of organ- and/or tissue-absorbed doses.

To date, little information has been reported regarding electrocardiogram (ECG)-gated scanning-based dose assessment methods.^(19)^ Although one of the primary CT dose assessment software as the Imaging Performance Assessment of CT scanner (ImPACT) patient dosimetry version 1.04 software (released in May 2011; Scanner Evaluation Centre of the United Kingdom National Health Service), could not be estimated the CCTA dose.^(20)^

The primary objective of this study was to assess radiation doses using modified ImPACT for cardiac applications [e.g., Ca score, target CTA, prospective CTA, continuous CTA/cardiac function analysis (CFA), and CTA/CFA modulation], as described by Kobayashi,^(20)^ on an Aquilion ONE ViSION Edition scanner (Toshiba Medical Systems, Otawara, Japan). For these processes, we clarified the CTDI to determine the relationship between HR and X-ray exposure when using cardiac applications. As a secondary objective, we compared radiation doses determined using modified ImPACT, a whole-body dosimetry phantom study, and the k-factor study to evaluate the practicality of modified ImPACT.

**II. Methods**

**A. C.F. evaluation**

A multi-detector-row CT (MDCT) scanner with 320 rows of detector elements (320-MDCT, Aquilion ONE ViSION Edition), which is capable of data acquisition at a slice thickness of 0.5 mm and coverage of 160 mm, was used in this study. The applicable CTDI for each of the scan protocols was obtained from the CT console display, as summarized in Table 1. The C.F. was then determined according to the following equation:

(1),


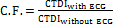

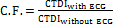


where CTDI_without ECG_ is the standard CTDI with a single rotation scan, as defined by a number of international organizations,^(11-17)^ and CTDI_with ECG_ is the CTDI normalized to a single-rotation ECG-gated scanning of the cardiac applications (Ca score, target CTA, prospective CTA, continuous CTA/CFA, and CTA/CFA modulation).

**B. Cardiac applications**

320-MDCT has cardiac applications of the prospective ECG-triggering scans (Ca score, target CTA, and prospective CTA) and retrospective ECG reconstruction scans (continuous CTA/CFA and CTA/CFA modulation) as B.1 to B.5. Completely different scan techniques are used in accordance with the patient’s HR and the purposes to reduce their radiation dose. It is common knowledge that these scan timing of scan techniques have relation to heart rate (HR) closely. According to previous publications, the optimal cardiac phase is generally 75% of the R-R interval (late diastole) for HR 40–60 beats per minute (bpm) or 40% (end systole) for 70–120 bpm.^(8-10)^ When HR was increased, the T-P interval gradually be shortened therefore, coronary only has low motion in the interval of late diastole (T-P interval) at low HR beats and end systole (Q –T interval) at high HR beats. In this reason, an HR gradient of 40–120 bpm in 1-bpm increments was used to estimate CTDI_with ECG_, except for modulation, for which an HR gradient of 40–80 in 1-bpm increments was used. Because of the modulation did not work in high HR. All CTDI_with ECG_ values were obtained from the CT console display.

**B.1 Ca score**

The Ca score (a Framingham risk score) is an effective index for determining the appropriate program to treat the amount of Ca in coronary arteries. This is a low-dose scanning technique involving the exposure time and a single cardiac phase. Therefore, CTDI_with ECG_ is obtained during the prespecified phases of late diastole (40–70 bpm or 60–120 bpm).

**B.2 Target CTA**

Target CTA is a low-dose scanning technique in which the exposure time and a single cardiac phase are manually preset before scanning to ensure that the patient receives consistent exposure. The actual exposure time depends on the active time (i.e., absolute time; 0.275, 0.3, 0.35, and 0.4 s). Therefore, this scan mode cannot correspond to arrhythmia. CTDI_with ECG_ for this method is obtained during the same as phase as the Ca score according to the active time.

**B.3 Prospective CTA**

Prospective CTA is a low-dose scanning technique in which exposure occurs only during the prespecified range of the cardiac phase.^(8,9)^ Therefore, the actual exposure time varies according to the patient’s HR. In addition, multi-segmental reconstruction is available for patients with high HRs in whom multiple beats are scanned. With prospective CTA, CTDI_with ECG_ is obtained during the prespecified cardiac phase range of 1%–99% in 1% increments (i.e., all cardiac phase ranges, but less than a continuous scan).

**B.4 Continuous CTA/CFA**

Continuous CTA/CFA is a scanning technique in which exposure occurs throughout the R-R interval over one or more heartbeats. Therefore, a functional analysis can be performed using the obtained data. Similar to prospective CTA, multi-segmental reconstruction is available for patients with high HRs in whom multiple beats are scanned. In this scan mode, CTDI_with ECG_ is obtained throughout the cardiac cycle (0%–100%; R-R interval). In general, only data acquired during the cardiac phase with the least amount of motion are used for image reconstruction.

**B.5 CTA/CFA Modulation**

CTA/CFA modulation is a scanning technique in which exposure occurs throughout the R-R interval and over one or more heartbeats. Exposure is also available to reduce the mA during portions of the R-R interval that do not require high-resolution imaging. Therefore, CTDI_with ECG_ is obtained as follows: the tube current decreases automatically, except for the prespecified dose increase phase of 1%–99%. In this case, the 100% phase was not estimated because the tube current was fixed at that point. The dose reduction rate ranges from 5% to 80%. The C.F. is subsequently calculated using equation (1).

**C. ImPACT patient dosimetry**

ImPACT patient dosimetry spreadsheet software, version 1.0.4 for Excel (Microsoft Corp., Redmond WA, USA), was developed by the ImPACT group to provide a convenient user interface for determining organ- and tissue-absorbed doses according to the National Radiological Protection Board SR250 Monte Carlo dose data sets (NRPB-SR250).^(21,22)^ ImPACT reflects the further development of a method to map results from the original 23-scanner data sets to other CT scanners by applying so-called “ImPACT factors” on the basis of tube voltage-dependent CTDI in free air (CTDI_air_) and CTDI in the center (CTDI_100,c_) with either a standard head or standard body polymethylmethacrylate (PMMA) phantom. The Medical International Radiation Dose (MIRD)-5 mathematical phantom used in ImPACT was divided from head to mid-thigh into 208 axial slabs of 5-mm thick.^(23)^

General usage for assessing the radiation dose (e.g., CTDI, DLP, organ/tissue absorbed dose, effective dose) was determined according to the following parameters: CT scanner, tube voltage, tube current, rotation time, spiral pitch, collimation, scan range, scan region (head or body), and organ weighting scheme, as described in ICRP 60^(24)^ or 103.^(25)^

A checkbox, combo boxes, and text boxes were added to allow the selection of cardiac applications in the ImPACT ScanCalculation worksheet (Fig. 1). If the checkbox was active, CTDI_with ECG_ was assessed according to the following equation:

(2),


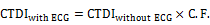

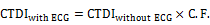


where CTDI_without ECG_ is the assessment results of original ImPACT. To assess the CTDI_without ECG_, the following protocols were added: scan mode, HR, cardiac phase range, and scan heartbeats. In some scan modes, acquisition time, dose reduction, and dose reduction range were necessary. The CTDI_w_, CTDI_vol_, and DLP dose assessments were then weighted using the C.F. reference lookup table and a newly created C.F. worksheet to obtain the C.F.-weighted absorbed doses and effective dose.

The effective dose was defined in ICRP publ.103,^(24)^ which used a more realistic description of the human body in the form of a voxel model phantom^(25)^ constructed from medical imaging data of a real person. However, the effective dose from ImPACT was estimated using the MIRD-5 phantom. Therefore, the reference phantom C.F., which links the MIRD-5 phantom to the ICRP 110 reference phantom,^(24)^ was used.^(20)^ The following concept was used to determine the reference phantom C.F.:

(3)


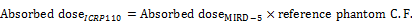

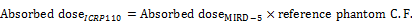


(4)


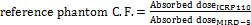

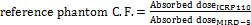


The reference phantom C.F. includes 14 CT examination categories (chest, chest–pelvis, abdomen–pelvis, abdomen, pelvis, adrenals, liver, kidneys, liver–kidneys, kidneys–bladder, head, neck, and head–neck). During CCTA examination, we considered the chest C.F. to be appropriate. In addition, we added evaluations of accumulated radiation doses for the plane and contrast enhancement scans to the existing worksheets containing scan protocols, scan areas, and dose results (Fig. 1).

**D. Input of CCTA scanning protocols**

The organ- and tissue-absorbed doses and effective dose were estimated for the CCTA scanning protocols (Tables 2 and 3) to confirm the software reliability. These protocols included the following: tube currents of 90 mA for plane scans (target CTA) and 450 mA for contrast enhancement scans (prospective or continuous CTA), a scan length of 128 mm, and a bolus tracking scan time of 2.7 s. Average data were obtained from 50 patients (mean age, 69 ± 9.6 years; height, 161.5 ± 9.2 cm; weight, 62.4 ± 11.7 kg). The bolus tracking time was calculated as the total time of the intermittent scans and continuous scan. However, 320-MDCT is incapable of stopping the continuous scan automatically within 10 s for mechanical restriction. Therefore, we performed four intermittent scans (2.55 s total) to provide stable reproducibility of the bolus tracking scan time, as a continuous scan with a manual stop is not stably reproducible. The scan areas were then determined as shown in Fig. 1. Note that a positioning scan was excluded from this assessment.

**E. Comparison of radiation doses**

TLDs were calibrated using an ionization chamber with a volume of 6 cm^3^ (10X5–6; Radcal Corporation, Monrovia, CA, USA) and a dosimeter (9015; Radcal) that was annually calibrated by a standard dosimetry laboratory. TLDs were then calibrated at an air kerma of 10 mGy from an effective energy of 54.6 keV [half-value layer (HVL) of aluminum (99.9 %); 7.88 mmAl] using the above-described ionization chamber and diagnostic X-ray equipment (KXO-81; Toshiba Medical Systems, Otawara, Japan) with an X-ray tube aluminum filter (DRX-3724HD; 1.1 mmAl: Toshiba Medical Systems, Otawara, Japan), collimation-filter (TF-6TL-6; 1.2 mmAl: Toshiba Medical Systems, Otawara, Japan), and additional 6.0 mmAl filter (purity: 99.9%).

The phantom study used a human body phantom in which molded polymer shapes, intended to simulate bone, were embedded in a material equivalent to soft tissue (Alderson Rando phantom with or without breasts; 175 cm, 73.5 kg; The Phantom Laboratory, Salem, NY, USA); 233 TLD elements (MSO-S; Kyokko, Japan) were additionally inserted into this phantom. The Rando phantom was placed in the supine position on a 320-MDCT table and irradiated to evaluate the radiation doses from CCTA (Tables 2 and 3). An individual C.F. was subsequently applied after measuring the amount of fluorescence (M) with a TLD reader (Model 3000; Kyokko, Hiroshima, Japan). The air-absorbed dose D_air_ was calculated from the fluorescence and C.F. (f) as indicated in equation (5):

(5)


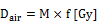

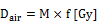


The sex-averaged organ- and tissue-absorbed dose D (Gy) was calculated using D_air_ and the ratio of the air and organ mass energy-absorption coefficient (μ_en_/ρ) at an effective energy of 54 keV, as follows:

(6)


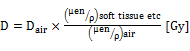

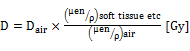


The sex-averaged equivalent dose H_T_ (Sv) was then calculated using D and a radiation weighting factor (1.0) specified in the ICRP publication 103.^(25)^

[Sv] (7)


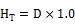

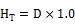


The effective dose E (Sv) was calculated using H_T_ and the age- and sex-averaged weighting factor W_T_ for each organ or tissue:^(25)^

(8)


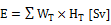

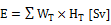


Regarding the remaining tissues and organs, the adrenal gland, gallbladder, heart, kidney, pancreas, prostate gland (for males), small intestine, spleen, and uterus (for females) were evaluated. The endosteal bone surface dose enhancement factors referenced by Nishizawa for each bone type were used.^(27)^

In contrast, the effective dose was estimated according to the following equation for comparison with the k-factor study; specifically, the effective dose was calculated from the DLP displayed on the CT console and the k-factor for adult chest (0.014 mSv·mGy^−1^·cm^−1^) from the 2004 European Commission guidelines:^(13)^

(9)


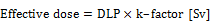

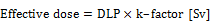


In addition, the effective dose was estimated using the C.F. values reported by Andrew^(6)^ useful for coronary evaluation, as follows: volume scan, 0.031 mSv·mGy^−1^·cm^−1^, and bolus tracking, 0.017 mSv·mGy^−1^·cm^−1^.

**III. Results**

**A. C.F. evaluation**

We first determined the C.F.s of cardiac applications (Ca score, target CTA, prospective CTA, continuous CTA/CFA, and CTA/CFA Modulation) according to equation 1 (Fig. 2–6). Fig. 2 demonstrates that the Ca score C.F. is independent of the cardiac phase and HR. The C.F. increased with longer X-ray tube rotation times. Fig. 3 demonstrates that the target CTA C.F. at an active time of 0.275 s was the same as the Ca score C.F. In addition, other C.F. values increased linearly with an increasing active time. Therefore, the target CTA C.F. remains independent of the cardiac phase and HR. Fig. 4 shows a correlation of the prospective CTA C.F. with HR and cardiac phase ranges. The C.F. tended to increase as the cardiac phase range widened. In addition, we clarified a limitation of the C.F. with respect to determining the cardiac phase range necessary for reducing the CTDI. Accordingly, the cardiac phase limitation was characterized by HR (10% at 60 bpm, 15% at 80 bpm, and 20% at 100 bpm). To reduce the patient dose, the cardiac phase range should be kept as narrow as possible. If the pre-specified narrow cardiac phase range exceeds these values, the patient dose will not be reduced. Fig. 5 shows that the continuous CTA/CFA C.F., which increased as HR decreased, was one to four times higher than that of the C.F. value obtained for the Ca score in Fig. 1. Fig. 6 shows the CTA/CFA modulation at a dose reduction of 50% C.F. in the 70%–90% dose cardiac phase range was the same as the value obtained for continuous CTA/CFA. At 80 bpm, even the 50% cardiac phase range was equivalent to the value obtained for continuous CTA/CFA. The CTA/CFA modulation at a dose reduction of 50% reduced the dose in comparison with that at a dose reduction of 25%. The cardiac phase range had less of an influence on the dose as the dose reduction decreased. In other words, this value also increased as the dose increase phase range increased. A reference table of cardiac application data was created from these data.

**B. Comparison of radiation doses**

Table 4 shows the organ doses and the effective doses estimated by the ImPACT study and phantom study. Within the same examination, some organs received noticeably different doses between phantoms. For example, the doses to the breasts in the reference male phantom (10.66 mGy at 60 bpm and 75.77 mGy at 90 bpm) were less than approximately 60% of the absorbed doses in the reference female phantom (17.50 mGy and 123.50 mGy). In contrast, the doses to the breasts for the reference person (14.08 mGy and 99.64 mGy) were similar to those in the phantom study (14.15 mGy and 101.66 mGy). In the CCA examination, the breasts had the highest absorbed doses among all the organs. Similarly, the absorbed doses to the lung were high because this organ was directly irradiated during the bolus tracking scan (Fig. 1). Regarding the lung absorbed dose, the ImPACT study doses were 8.72 mGy at 60 bpm and 62.48 mGy at 90 bpm vs. 11.20 mGy and 78.88 mGy at 60 and 90 bpm, respectively, during the phantom study. After the breasts and lung, the highest absorbed doses were observed in the stomach, liver, and esophagus because these organs were partially irradiated by volume scans. Therefore, doses absorbed by these organs were much higher than those absorbed by non-irradiated organs, such as the colon, gonads, bladder, thyroid, brain, and salivary glands. Furthermore, the absorbed doses to the lung, stomach, and liver according to ImPACT differed from those obtained in the phantom study. The absorbed doses on the bone surface (3.42 mGy and 24.62 mGy vs. 1.42 mGy and 11.26 mGy) were approximately two-fold higher than those obtained in the phantom study; however, the doses absorbed by the bone surface and bone marrow may have been inaccurate because of unclear tissue-absorbed dose measurements.

The effective doses for the reference male phantom (4.04 mSv at 60 bpm and 29.03 mSv at 90 bpm) were less than approximately 25% of those for the reference female phantom (5.28 mSv and 37.84 mSv), and the doses for the reference person (4.66 mSv and 33.43 mSv) were less than approximately 10% of those for the phantom study (5.28 mSv and 36.68 mSv), with differences of 0.62 mSv (13%) and 3.25 mSv (10%), respectively (Table 5). The effective doses obtained with the k-factor (0.014 mSv·mGy^−1^·cm^−1^; 2.57 mSv at 60 bpm and 17.10 mSv at 90bpm) were much lower than those obtained with the other two types of measurements; for example, the differences from ImPACT were 2.09 mSv (45%) and 16.33 mSv (50%), respectively. In contrast, the effective doses obtained with the C.F. values reported by Andrew (volume scan and bolus tracking scan at 0.031 and 0.017 mSv·mGy^−1^·cm^−1^, respectively; 5.65 mSv at 60 bpm and 37.81 mSv at 90 bpm) were more similar to those of the phantom study.

Finally, the newly designed worksheets (saved dose data, saved scan position) were useful for comparing radiation doses and confirming scan areas.

**IV. Discussion**

In this study, we have shown that modified ImPACT, when used to evaluate CCTA with cardiac applications (Ca score, target CTA, prospective CTA, continuous CTA/CFA, and CTA/CFA modulation), can assess radiation doses (CTDI, DLP, organ doses, and effective dose). We have additionally demonstrated that the CTDI can be characterized by cardiac applications (CTDI_with ECG_). These results suggest that dose assessment with modified ImPACT is a practical method for CCTA evaluation.

In many dose estimations involving CCTA cases, the k-factor (adult chest; 0.014 mSv·mGy^−1^·cm^−1^) is used.^(2)^ However, we thought that the effective dose would be underestimated because the chest k-factor is obtained from a portion of the entire chest rather than the heart itself. The adult coronary k-factors were introduced by Andrew^(6)^ and Gosling to assess the CCTA effective dose^(27)^; these authors reported C.F. values of 0.027–0.034 and 0.028 mSv·mGy^−1^·cm^−1^, respectively. Although these adult coronary k-factors can assess the effective dose, they cannot assess the organ- and tissue-absorbed doses. Therefore, the effective dose for CCTA is possibly underestimated when the adult chest k-factor is used. In contrast, the absorbed doses are assessed easily with modified ImPACT.

An earlier study by Kobayashi et al.^(20)^ described a method combining both scanner and phantom data. Although that study^(20)^ provided evidence of the considerable flexibility of ImPACT, it still failed to demonstrate a correspondence with CCTA dose assessments. Therefore, the present study used C.F.-based methodology to assess CCTA radiation doses. The obtained C.F. results indicate that each cardiac application has particular dose characteristics, which could be explained by the following: 1) prospective ECG-triggering scans (Ca score, target CTA, and prospective CTA) are active only during a prespecified cardiac phase within the cardiac cycle and 2) retrospective ECG reconstruction scans (continuous CTA/CFA and CTA/CFA modulation) are active throughout the entire cardiac cycle. Therefore, during Ca scoring, X-ray irradiation is only active for one X-ray tube rotation to obtain image data during a prespecified cardiac phase; therefore, the Ca scoring dose (CTDI_with ECG_) is independent of the cardiac phase and HR. Target CTA features a similar X-ray irradiation activation pattern, and the resulting dose increases linearly as the acquisition time increases. During prospective CTA, X-ray irradiation is active for one or more X-ray tube rotations because the prespecified cardiac phase time exceeds a single rotation time, and the resulting dose increases at low HRs with a wide prespecified cardiac phase range because the absolute time increases. However, in this study, we clarified a limitation of the prospective CTA dose when determining the cardiac phase range required for dose reduction. Therefore, narrow cardiac phase specification may not lead to dose reduction. During continuous CTA/CFA, X-ray irradiation remains active throughout the cardiac cycle, resulting in a much larger dose than that in other ECG-gated scans. The CTA/CFA modulation dose over a wide cardiac phase range of 70%–90% is similar to the continuous CTA/CFA dose because the dose reduction phase time is too short to modulate the tube current. We confirm that the results of the present study are useful for determining scanning protocols in ECG-based examinations. Therefore, these results were subsequently used for the C.F. (equation 2).

One limitation of modified ImPACT is that it reduces radiosensitive organs to simple geometric shapes. Therefore, the doses absorbed by the lung, stomach, and liver differed from those measured in the Rando phantom study. According to ImPACT, the doses absorbed by the bone surface were two-fold higher than those obtained in the phantom study. Although the tissue-absorbed doses in the phantom study were estimated using the method reported by Nishizawa,^(27)^ these doses may have been inaccurate because tissue-absorbed dose estimation has not been well reported.

Modified ImPACT yields a more accurate effective dose when compared with the k-factor method. In addition, the effective dose for the reference male phantom was less than approximately 25% of the dose for the reference female phantom. We confirm that this difference is due to differences in the dose absorbed by the breasts. In contrast, the effective doses for the reference phantom were less than approximately 10% of those obtained in the phantom study. These results occurred because of differences in factors such as phantoms, organ locations, TLD sensitivity, TLD insert positions, and estimated tissue-absorbed doses. For example, the effective dose was approximately 7.5-fold higher at 90 bpm than at 60 bpm. However, the continuous CTA/CFA 3-beat dose at 90 bpm was 8.4 (Fig. 5) because of over-beaming during each intermittent single-beat scan.

Finally, we consider the credibility of the C.F. values reported by Andrew. As reported in that seminal study, the heart C.F. is approximately two-fold the adult chest k-factor. The present study, however, shows that modified ImPACT and Andrew’s method identified similar effective doses. The greatest advantage of our study is the ability to perform absorbed dose. However, a potential weakness of the present study is that C.F. values were obtained only for the Aquilion ONE ViSION Edition scanner; therefore, we cannot extrapolate our findings to other scanners. In addition, the present study indicates the importance of an appropriate scan area when using the k-factor method, a problem that was previously noted by Shrimpton. Using CCTA, we speculated that in comparison to the k-factor, the effective doses to the head or neck (orbit, middle ear, teeth, and face), chest (heart and breasts), and abdomen (liver and kidneys) would differ. Therefore, further studies of the k-factor are needed to assess the effective doses for all types of examinations.

**V. Conclusions**

The present study demonstrated that modified ImPACT could perform ECG-gated scanning-based radiation dose assessments and may thus be useful in future applications.

**Acknowledgment**

Redacted to preserve anonymity of review process

**References**

1. The Japanese Registry of All Cardiac and Vascular Diseases [in Japanese]. 2015. Retrieved February 20, 2015, from; <http://jroadinfo.ncvc.go.jp/?page_id=13>
2. United Nations Scientific Committee on the Effects of Atomic Radiation. UNSCEAR 2008 report to the General Assembly with Scientific Annexes. VOLUME I. New York: United Nations; 2010.
3. [Hunold P](http://www.ncbi.nlm.nih.gov/pubmed?term=Hunold%20P%5BAuthor%5D&cauthor=true&cauthor_uid=12511683), [Vogt FM](http://www.ncbi.nlm.nih.gov/pubmed?term=Vogt%20FM%5BAuthor%5D&cauthor=true&cauthor_uid=12511683), [Schmermund A](http://www.ncbi.nlm.nih.gov/pubmed?term=Schmermund%20A%5BAuthor%5D&cauthor=true&cauthor_uid=12511683), et al. Radiation exposure during cardiac CT: effective doses at multi-detector row CT and electron-beam CT. Radiology 2003;226(1):145-152.
4. [Mori S](http://www.ncbi.nlm.nih.gov/pubmed?term=Mori%20S%5BAuthor%5D&cauthor=true&cauthor_uid=17628377), [Nishizawa K](http://www.ncbi.nlm.nih.gov/pubmed?term=Nishizawa%20K%5BAuthor%5D&cauthor=true&cauthor_uid=17628377), [Kondo C](http://www.ncbi.nlm.nih.gov/pubmed?term=Kondo%20C%5BAuthor%5D&cauthor=true&cauthor_uid=17628377), [Ohno M](http://www.ncbi.nlm.nih.gov/pubmed?term=Ohno%20M%5BAuthor%5D&cauthor=true&cauthor_uid=17628377), [Akahane K](http://www.ncbi.nlm.nih.gov/pubmed?term=Akahane%20K%5BAuthor%5D&cauthor=true&cauthor_uid=17628377), [Endo M](http://www.ncbi.nlm.nih.gov/pubmed?term=Endo%20M%5BAuthor%5D&cauthor=true&cauthor_uid=17628377). Effective doses in subjects undergoing computed tomography cardiac imaging with the 256-multislice CT scanner. Eur J Radiol. 2008;65(3):442-448.
5. [Einstein AJ](http://www.ncbi.nlm.nih.gov/pubmed?term=Einstein%20AJ%5BAuthor%5D&cauthor=true&cauthor_uid=17635892), [Henzlova MJ](http://www.ncbi.nlm.nih.gov/pubmed?term=Henzlova%20MJ%5BAuthor%5D&cauthor=true&cauthor_uid=17635892), [Rajagopalan S](http://www.ncbi.nlm.nih.gov/pubmed?term=Rajagopalan%20S%5BAuthor%5D&cauthor=true&cauthor_uid=17635892). Estimating risk of cancer associated with radiation exposure from 64-slice computed tomography coronary angiography. JAMA. 2007;298(3):317-323.
6. [Einstein AJ](http://www.ncbi.nlm.nih.gov/pubmed?term=Einstein%20AJ%5BAuthor%5D&cauthor=true&cauthor_uid=18371595), [Sanz J](http://www.ncbi.nlm.nih.gov/pubmed?term=Sanz%20J%5BAuthor%5D&cauthor=true&cauthor_uid=18371595), [Dellegrottaglie S](http://www.ncbi.nlm.nih.gov/pubmed?term=Dellegrottaglie%20S%5BAuthor%5D&cauthor=true&cauthor_uid=18371595), [et](http://www.ncbi.nlm.nih.gov/pubmed?term=Rajagopalan%20S%5BAuthor%5D&cauthor=true&cauthor_uid=18371595) al. Radiation dose and cancer risk estimates in 16-slice computed tomography coronary angiography. J Nucl Cardiol. 2008;15(2):232-240.
7. CT Imaging Users Guide ImPACTDose 2.1 Document Version 1.1. Retrieved February 20, 2015, from <http://www.ct-imaging.de/images/Downloads/impactdose_usermanual.pdf>
8. [Abbara S](http://www.ncbi.nlm.nih.gov/pubmed?term=Abbara%20S%5BAuthor%5D&cauthor=true&cauthor_uid=19409872), [Arbab-Zadeh A](http://www.ncbi.nlm.nih.gov/pubmed?term=Arbab-Zadeh%20A%5BAuthor%5D&cauthor=true&cauthor_uid=19409872), [Callister TQ](http://www.ncbi.nlm.nih.gov/pubmed?term=Callister%20TQ%5BAuthor%5D&cauthor=true&cauthor_uid=19409872), [et](http://www.ncbi.nlm.nih.gov/pubmed?term=Weigold%20WG%5BAuthor%5D&cauthor=true&cauthor_uid=19409872) al. SCCT guidelines for performance of coronary computed tomographic angiography: A report of the Society of Cardiovascular Computed Tomography Guidelines Committee. J Cardiovasc Comput Tomogr. 2009;3(3):190-204.
9. Raff GL, Abidov A, Achenbach S, et al. SCCT Guidelines for the Interpretation and Reporting of Coronary Computed Tomographic Angiography. J Cardiovasc Comput Tomogr. 2009;3(2):122-136.
10. [Halliburton SS](http://www.ncbi.nlm.nih.gov/pubmed?term=Halliburton%20SS%5BAuthor%5D&cauthor=true&cauthor_uid=21723512), [Abbara S](http://www.ncbi.nlm.nih.gov/pubmed?term=Abbara%20S%5BAuthor%5D&cauthor=true&cauthor_uid=21723512), [Chen MY](http://www.ncbi.nlm.nih.gov/pubmed?term=Chen%20MY%5BAuthor%5D&cauthor=true&cauthor_uid=21723512), et al. SCCT guidelines on radiation dose and dose-optimization strategies in cardiovascular CT. J Cardiovasc Comput Tomogr. 2011;5(4):198-224.
11. ICRP Publication 87. Managing patient dose in computed tomography. Ann ICRP. 2000.
12. ICRP Publication 102. Managing patient dose in multi-detector computed tomography (MDCT). Ann ICRP. 2007.
13. Bongartz G, Golding SJ, Jurik AG, et al. European Guidelines on Quality Criteria for Computed Tomography. EUR 16262. The European Commission’s Study Group on Development of Quality Criteria for Computed Tomography. Luxembourg, Luxembourg: European Commission, 2004.
14. Bongartz G, Golding SJ, Jurik AG, et al. European Guidelines on Quality Criteria for Computed Tomography. EUR 16262. The European Commission’s Study Group on Development of Quality Criteria for Computed Tomography. Luxembourg, Luxembourg: European Commission, 2000.
15. International Electrotechnical Commission. International standard of IEC 60601-2-44 Ed1 Amendment 1: Medical electrical equipment–Part 2-44: Particular requirements for the safety of X-ray equipment for computed tomography. Geneva, Switzerland: International Electrotechnical Commission, 2000.
16. International Electrotechnical Commission. International standard of IEC 60601-2-44 Ed2 Amendment 1: Medical electrical equipment–Part 2-44: Particular requirements for the safety of X-ray equipment for computed tomography. Geneva, Switzerland: International Electrotechnical Commission, 2003.
17. International Electrotechnical Commission. International standard of IEC 60601-2-44 Ed3 Amendment 1: Medical electrical equipment–Part 2-44: Particular requirements for the safety of X-ray equipment for computed tomography. Geneva, Switzerland: International Electrotechnical Commission, 2009.
18. [Einstein AJ](http://www.ncbi.nlm.nih.gov/pubmed?term=Einstein%20AJ%5BAuthor%5D&cauthor=true&cauthor_uid=20177085), [Elliston CD](http://www.ncbi.nlm.nih.gov/pubmed?term=Elliston%20CD%5BAuthor%5D&cauthor=true&cauthor_uid=20177085), [Arai AE](http://www.ncbi.nlm.nih.gov/pubmed?term=Arai%20AE%5BAuthor%5D&cauthor=true&cauthor_uid=20177085), et al. Radiation dose from single-heartbeat coronary CT angiography performed with a 320-detector row volume. Radiology. 2010;254(3):698-706.
19. Kobayashi M, Asada Y, Matsubara K, et al. Evaluation of the CT dose index for scans with an ECG using a 320-row multiple detector CT scanner. Radiat Prot Dosimetry. 2014 Nov 11. doi:10.1093/rpd/ncu331.
20. Kobayashi M, Asada Y, Matsubara K, et al. Evaluation of organ doses and effective dose according to the ICRP Publication 110 reference male/female phantom and the modified ImPACT CT patient dosimetry. J Appl Clin Med Phys. 2014;15(5):1-11.
21. Impactscan.org. London, UK. Retrieved Feb 20, 2015, from <http://www.impactscan.org>.
22. Jones DG, Shrimpton PC. Normalised organ doses for x-ray computed tomography calculated using Monte Carlo techniques. NRPB Report SR250. Didcot, Oxfordshire, England: National Radiological Protection Board, 1993.
23. Brownell GL, Ellett WH, Reddy AR. Absorbed fractions for phantom dosimetry. MIRD Pamphlet No. 3. J Nucl Med. 1968;9:27–39.
24. ICRP Publication 60. 1990 Recommendations of the International Commission on Radiological Protection. Ann ICRP. 1991.
25. ICRP Publication 103. The 2007 Recommendations of the International Commission on Radiological Protection. Ann ICRP. 2007.
26. ICRP Publication 110. Adult Reference Computational Phantoms. Ann ICRP. 2009.
27. Nishizawa K, Maruyama T, Takayama M, Iwai K, and Furuya Y. Estimation of effective dose from CT examination. Jpn J Radiol. 1995;55(11):763–768.

Fig. 1. Representative examples of the ECG-gated scan mode, saved dose data, saved position data, and scanning range of the CCTA examination. The MIRD-5 mathematical phantom was divided from head to mid-thigh into 208 axial slabs of 5-mm thickness. The CCTA scanning area is shown on the phantom.

Fig. 2. CTDI with ECG-gated scanning for the Ca score.CTDI was obtained from the CT console display for each of the scan protocols. The cardiac phase is 75% of the R-R interval (late diastole) for 40–60 bpm and 40% (end systole) for 70–120 bpm.

Fig. 3. CTDI with ECG-gated scanning for target CTA. CTDI was obtained from the CT console display for each of the scan protocols. The tube rotation time was 0.275 s/rot.

Fig. 4. CTDI with ECG-gated scanning for prospective CTACTDI was obtained from the CT console display for each of the scan protocols. C.F. is limited when determining the cardiac phase range required to reduce the CTDI (10% at 60 bpm, 15% at 80 bpm, and 20% at 100 bpm). If the narrow, pre-specified cardiac phase range exceeds these values, the patient dose is not reduced.

Fig. 5. CTDI with ECG-gated scanning for continuous CTA/CFA. CTDI was obtained from the CT console-display for each of the scan protocols. The C.F. was increased with decreasing HR.

Fig. 6. CTDI with ECG-gated scanning for CTA/CFA modulation. CTDI was obtained from the CT console-display for each of the scan protocols. One heartbeat is short, tube current is not modulated for HR of 80 bpm or more.

Table 1. Scan protocols used for the CTDI C.F.

Table 2. Scan protocol for a coronary CT examination at 60 bpm


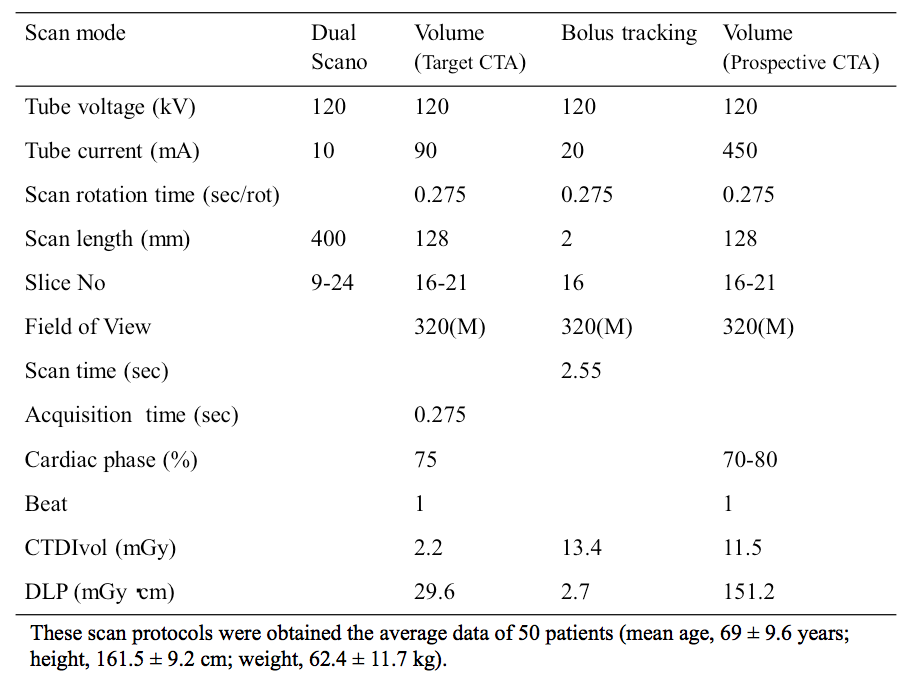


Table 3. Scan protocol for a coronary CT examination at 90 bpm


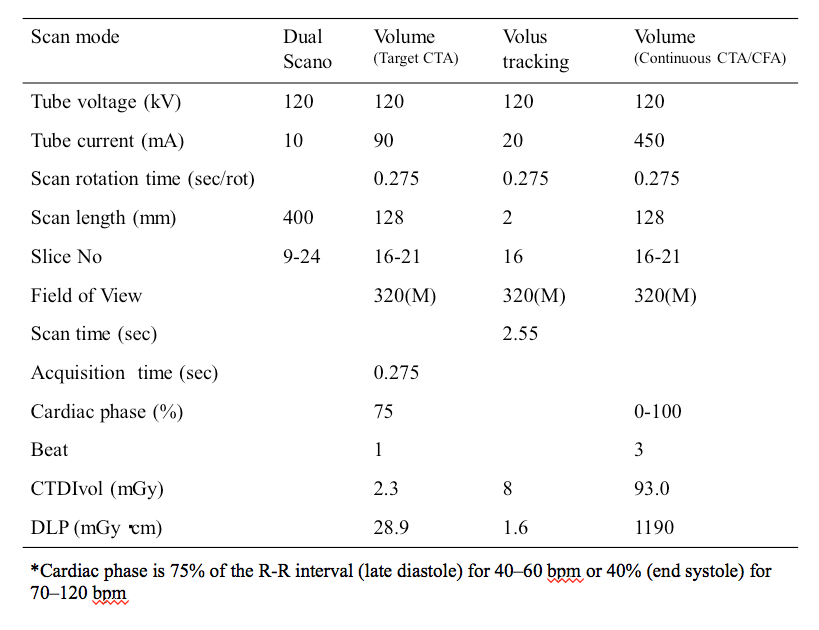


Table 4. Comparisons between different organ dose measurements

Table 5. Comparisons between different effective dose measurements


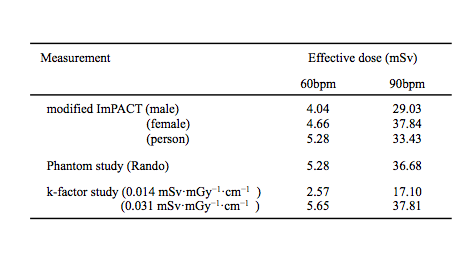

Supplement: Supplementary file 1 — Supplementary Material [file ACM2-17-342-s001.docx]
